# Parental and perinatal risk factors for sexual offending in men: a nationwide case-control study

**DOI:** 10.1017/S003329171600249X

**Published:** 2016-10-13

**Authors:** K. M. Babchishin, M. C. Seto, A. Sariaslan, P. Lichtenstein, S. Fazel, N. Långström

**Affiliations:** 1 Royal's Institute of Mental Health Research, University of Ottawa, Ottawa, Canada; 2 Department of Medical Epidemiology and Biostatistics, Karolinska Institutet, Stockholm, Sweden; 3 Department of Psychiatry, University of Oxford, Oxford, UK; 4 Department of Neuroscience, Uppsala University, Sweden

**Keywords:** Case-control, parental characteristics, perinatal, risk factor, sex offending

## Abstract

**Background:**

Prior studies suggest parental and perinatal risk factors are associated with later offending. It remains uncertain, however, if such risk factors are similarly related to sexual offending.

**Method:**

We linked socio-demographic, family relations, and perinatal (obtained at birth) data from the nationwide Swedish registers from 1973 to 2009 with information on criminal convictions of cases and control subjects. Male sex offenders (*n* = 13 773) were matched 1:5 on birth year and county of birth in Sweden to male controls without sexual or non-sexual violent convictions. To examine risk-factor specificity for sexual offending, we also compared male violent, non-sexual offenders (*n* = 135 953) to controls without sexual or non-sexual violent convictions. Predictors included parental (young maternal or paternal age at son's birth, educational attainment, violent crime, psychiatric disorder, substance misuse, suicide attempt) and perinatal (number of older brothers, low Apgar score, low birth weight, being small for gestational age, congenital malformations, small head size) variables.

**Results:**

Conditional logistic regression models found consistent patterns of statistically significant, small to moderate independent associations of parental risk factors with sons’ sexual offending and non-sexual violent offending. For perinatal risk factors, patterns varied more; small for gestational age and small head size exhibited similar risk effects for both offence types whereas a higher number of older biological brothers and any congenital malformation were small, independent risk factors only for non-sexual violence.

**Conclusions:**

This nationwide study suggests substantial commonalities in parental and perinatal risk factors for the onset of sexual and non-sexual violent offending.

## Introduction

Sexual offending is a serious societal and public health problem (WHO, 2013). Attempts at preventing sexual offending will be most effective when based on a robust aetiological understanding. Studies suggest that parental and perinatal factors are causally related with later criminal behaviour (i.e. after accounting for familial confounding; Ellingson *et al.* 2012; Kuja-Halkola *et al.* 2012; Coyne *et al.* 2013*b*). For example, younger maternal age (Coyne *et al.* 2013*a*), lower maternal education (Kendler *et al.* 2014), paternal age (Kuja-Halkola *et al.* 2012), greater number of siblings (Kolvin *et al.* 1990), and parental illness (Kolvin *et al.* 1990) are associated with greater likelihood of offspring criminality. However, no study has rigorously examined whether such factors are associated with sexual offending.

It is parsimonious to view sexual offending as a manifestation of general antisocial tendencies because a substantial proportion of sex offenders also commit non-sexual offences (Babchishin *et al*. 2016). Some risk factors are also common for both sexual and non-sexual offending (Fazel *et al.* 2007; Kjellgren *et al.* 2010). At the same time, however, representative population surveys suggest that only a small proportion of those who commit non-sexual violence also commit a sexual offence (Kjellgren *et al.* 2010). This suggests that additional, unique risk factors for sexual offending are involved. In particular, excessive sexual preoccupation and paraphilias, such as paedophilia and exhibitionism, are more common in sex offenders than in non-sexual offenders and non-offending men (Seto, 2008, 2013; Whitaker *et al.* 2008). Paedophilia is not a necessary or sufficient condition for sexual offending, but approximately half of sexual offenders against children have paedophilia (Seto, 2008). Some have suggested that risk factors for paedophilia are present before birth (Seto, 2012). As such, perinatal factors could be especially informative in understanding the aetiology of sexual offending.

The extent to which parental and perinatal risk factors influence the onset of sexual offending is unclear. Sexual offending clusters in families (Långström *et al.* 2015), and is largely explained by genetic and unique, non-shared environmental factors. Retrospective studies of selected offender samples suggest that perinatal complications are more common among paedophilic sexual offenders than non-paedophilic groups (small to medium effects; Blanchard *et al.* 2000; Côté *et al.* 2002; Dyshniku *et al.* 2015; Poeppl *et al.* 2015). There is also evidence for a role for prenatal factors. For example, left-handedness is a prenatally determined characteristic, and paedophilic sexual offenders are three times more likely to be left-handed than non-paedophilic groups (Cantor *et al.* 2005). Men with a greater number of older brothers have been found to report more paraphilic sexual interest (*d* = 0.49; Rahman & Symeonides, 2007); suggesting a maternal immune sensitization process may be involved in paedophilia and, more specifically, sexual interest in male children (Blanchard & Bogaert, 1998; Blanchard *et al.* 2000; Quinsey, 2003).

With a few exceptions (e.g. Christofferson *et al.* 2005; Långström *et al.* 2015), studies on parental and perinatal risk factors for sexual offending have used cross-sectional or retrospective designs and smaller, selected samples referred to treatment centers. Although heuristically valuable, such studies are likely to be limited by offender recall and selection biases, limited power, and poor control over familial confounders, such as parental criminality.

### Present study

We investigated the effects of parental and perinatal factors on risk of sexual and non-sexual violent offending in a Swedish, nationwide, case-control study of over 37 years while accounting for possible bias of confounding variables. There are multiple possible factors that could have been examined using these registries (e.g. psychiatric diagnoses prior to the offence). We decided to focus on certain distal factors for the current study because of our interest in the earliest origins of sexual offending. Although there is also a wide range of parental and perinatal factors that could be examined, we were limited by availability of reliably collected variables in the linked datasets. Only men were studied because the low proportion of female sex offenders (2%) precluded sub-analyses with sufficient statistical power. To examine if potential risk factors were specific to sexual offending, we also conducted parallel analyses with offenders convicted of non-sexual violent offences.

## Method

### Study setting and case identification

Swedish population-based registries, with prospectively collected data, were linked using the unique personal identification number assigned to every Swedish resident. A 1:5 nested case-control design matched on birth year and county of birth was used to examine if risk factors were associated with sexual offending in men. Matching on birth year was done so that each case and their respective controls would have the same period at risk for committing an offence and similar likelihoods of being arrested, prosecuted, and convicted as reflected in national registers. Matching on birth year also controlled for any cohort effects in the prevalence of the examined risk factors or offending, or in their associations, over time, and addressed bias from left-truncation. We also matched for county of birth to manage any bias from regional variation in practices regarding documentation of the examined risk factors, data quality, and efficiency of the judicial system.

The study design required that all cases and controls had Swedish birth registry information; hence, all participants were born in Sweden. Cases had a conviction for any sexual offence according to the Swedish Penal Code, from 15 years of age (the age of criminal responsibility in Sweden). We used the first sexual offence conviction if there was more than one. Matched controls, five to each case, were not allowed to have any sexual or violent non-sexual conviction up until the offence date for the sex offender. Sexual offence included three main categories: (*a*) rape or sexual coercion against an adult; (*b*) intra- or extra-familial child sex offences; and (*c*) non-contact sexual offences such as child pornography offences, sexual harassment, or indecent exposure. Recognizing that our definition of sexual crime contained several perhaps aetiologically distinct subtypes (Seto, 2008, 2013), we also conducted analyses separately for sex offenders against adults and sex offenders against children, as well as sex offenders with non-sexual violent convictions and sex offenders without non-sexual violent convictions.

Finally, we carried out parallel analyses with non-sexual violent offenders to determine if tested risk factors were specific to sexual offending. Non-sexual violence was defined according to the Swedish Penal Code, and included homicide, assault, robbery, or illegal threats. Convictions for attempted or aggravated offences were also included whenever applicable (Frisell *et al.* 2011). For each violent non-sexual offender, five new controls were randomly selected from the men in the general population and matched on birth year and county of birth. Selected controls did not have any sexual or non-sexual violent conviction up until the offending date of the non-sexual violent offender. Non-sexual violent case individuals were not allowed to have a sexual offence, before or after the index non-sexual violent offence.

### Population

Participants included all male convicted sex offenders (*n* = 13 773) born in Sweden between 1973 and 1995 (median = 1983). Of these, 6263 sexually offended against children and 8584 against adults before the follow-up end date (2009); these two subgroups are not mutually exclusive and, hence, add up to more than 100%. Subgroup datasets could include those with both sexual offence types (*n* = 1074, 7.8% of the overall sample).

Age at first conviction ranged from 15 to 38 years [interquartile range (IQR)17–25]. Approximately one third of sex offenders had more than one sex crime conviction (one conviction, 66.2%, 2, 19.2%, 3, 6.2%, ⩾4, 8.4%; IQR 1–2). More than half of sex offenders also had a conviction for a non-sexual violent offence (55.4%, 7623/13 773). Sexual offenders were matched to 68 718 controls without a sexual or non-sexual violent offence conviction (also aged 15–38 years; IQR 17–25).

Violent non-sexual offenders (*n* = 135 953) were also born in Sweden between 1973 and 1995 (median 1983) and did not have a sexual offence conviction up until the follow-up end date (2009). Age at violent offence was defined as the age at first violent conviction, and ranged from 15 to 38 years (IQR 16–22). They were matched to 680 120 controls without a sexual or non-sexual violent conviction (also aged 15–38 years; IQR 16–22).

### Measures

#### Crime

The National Crime Register (held by the National Council for Crime Prevention) provided data on all criminal convictions for 1973–2009 (arrests or charges were not recorded in this register). According to Swedish Penal Law, offenders are convicted regardless of whether they have a mental illness; hence, the register includes those receiving ‘not guilty by reason of insanity’ verdicts and forensic psychiatric care, as well as non-custodial sentences, fines or cautions. Plea-bargaining is not permitted in Sweden, so sexually motivated offences were always registered as such.

#### Obstetric and birth data

The Medical Birth Register (National Board of Health and Welfare) has mandatory reporting and includes prospective data on more than 99% of births and preceding pregnancies in Sweden from 1973 onwards. We used *small head circumference* (defined as ⩽33 cm), *low birth weight* (defined as <2500 g), and being *small for gestational age* (defined as ⩾2 s.d. below the mean birth weight for gestational age; Marsal *et al.* 1996). *Congenital malformations* were coded according to the International Statistical Classification of Diseases (ICD; ICD-8/9: 760–779; ICD-10: P00-P99). A *low Apgar score at 5 min* was defined as <7. *Number of older brothers* was defined as the number of males the mother had given birth to prior to the birth of the case or control.

#### Parental characteristics

*Highest education* obtained by any of the biological parents (0, primary school; 1, secondary school; 2, post-secondary qualification) was collected from the Education Registry. Data on parental psychiatric morbidity requiring inpatient treatment before the son's birth were obtained from the National Patient Register, which holds data for all individuals discharged from every Swedish hospital since 1973. We coded any parental *major psychiatric disorder* (yes/no) as at least one of *psychotic* (schizophrenia spectrum and other non-organic psychoses but not bipolar disorder, ICD-8: 291, 295, 297, 298, 299; ICD-9: 295, 297, 298; ICD 10: F20-F25, F28-F29, F32.3, and x.5 in F10-F19), *affective* (depressive and bipolar disorders, ICD-8: 296.1, 296.0, 296.2–296.8, 300.4, ICD-9: 296A, 296B-296E, 296W, 296X, 300E, 311, ICD-10: F30-F39 except 32.3), or *personality disorder* (ICD-8/ICD-9: 301 and ICD-10: F60). *Any parental substance use disorder* (yes/no; ICD-8: 303, 304; ICD-9: 303, 305A, 305X; ICD-10: F10, except F10-F19 x.5) and *suicide attempt* (yes/no; ICD-8/ICD-9: E950-E959, E980-E989; ICD-10: X60-X84, Y10-Y34) were also coded. The number of violent (including violent sexual) convictions by parents was obtained from the National Crime Register and coded dichotomously (0, no parental conviction; 1, at least one parent had a violent conviction).

As seen in Supplementary Fig. S1, there were curvilinear relationships between paternal and maternal age and offspring sexual and non-sexual violent criminality, respectively. To reflect these observed relationships, *paternal and maternal age* were both recoded into an ‘under age 25 years’ category (reference category 25–44.9 years). In addition, we coded an ‘over age 45 years’ category for paternal age (reference category 25–44.9 years); however, since these two variables (young age and older age) were correlated >0.80 it was dropped from the analyses. Hence, only maternal and paternal age <25 years (*v.* 25–44.9 years) were included in the model.

### Statistical analyses

Conditional logistic regression, which is recommended for matched case-control designs (Hosmer *et al.* 2013), was used to examine the association between risk factors and subsequent offending by sons. Two independent models were examined, one for parental and one for perinatal risk factors. To address the risk of multicollinearity in multivariable analyses, we tested risk factor intercorrelations. Some were intercorrelated more strongly than ±0.70, the strongest being the association between young mother and young father at 0.79 (Supplementary Table S1). No risk factor was excluded from multivariable logistic regression models for this reason. The final multivariable models included all risk factors significant at *p* < 0.05 in bivariate analyses. The specificity of putative risk factors was tested by comparing their associations with both sexual and violent non-sexual offending, respectively. Non-overlapping 95% confidence intervals around point estimates indicated that differences between offence types were statistically significant at *p* < 0.01 (Tryon, 2001). All analyses were conducted using Stata v. 13 (StataCorp, 2013).

## Ethical statement

The authors assert that all procedures contributing to this work comply with the ethical standards of the relevant national and institutional committees on human experimentation and with the Helsinki Declaration of 1975, as revised in 2008.

## Results

### Sexual offending

In bivariate analyses (Table 1), all seven examined parental factors (model 1) – young parental ages at delivery, lower parental education, any parental violent conviction, psychiatric disorder, substance use disorder, and suicide attempt – were positively associated (*p* < 0.05) with sons’ sexual offending risk (small to moderate effects, according to Cohen, 1988). Among the six perinatal factors (model 2), low birth weight, being small for gestational age, and small head circumference were associated with increased risk of sexual offending (small to moderate effect sizes).

**Table 1.** Birth year, parental, and perinatal risk factors for any sexual offending in a Swedish nationwide case-control study over 37 years

Variables	% (*n/N*) or mean (s.d., *N*)		Unadjusted logistic regression		Adjusted logistic regression^a^	
	Sex offenders	Controls	OR	(95% CI)	aOR	(95% CI)
Birth year^b^	1983 (s.d. = 6.00, *n* = 13 773)	1983 (s.d. = 6.00, *n* = 68 718)	–	–	–	–
**Model 1: Parental risk factors (*N* = 76 636, *n*_sexual offenders_ = 13 168)**						
Age of mother at delivery <25 years^c^	42.0% (5719/13 607)	27.9% (19 074/68 393)	**1.89**	**(1.82–1.97)**	**1.42**	**(1.36–1.49)**
Age of father at delivery <25 years^c^	22.8% (3017/13 230)	13.8% (9254/66 985)	**1.85**	**(1.77–1.94)**	**1.24**	**(1.17–1.31)**
Highest parental education^d^	0.86 (s.d. = 0.54, *n* = 13 543)	1.09 (s.d. = 0.56, *n* = 68 288)	**0.48**	**(0.46–0.50)**	**0.56**	**(0.54–0.58)**
Any parental violent conviction	21.7% (2949/13 591)	6.7% (4553/68 362)	**3.93**	**(3.73–4.14)**	**2.97**	**(2.80–3.14)**
Any parental psychiatric disorder	4.7% (638/13 614)	2.1% (1423/68 438)	**2.33**	**(2.11–2.56)**	**1.53**	**(1.36–1.72)**
Any parental substance use disorder	3.6% (483/13 614)	1.1% (732/68 438)	**3.45**	**(3.07–3.89)**	**1.64**	**(1.42–1.91)**
Any parental suicide attempt	3.1% (426/13 614)	1.3% (918/68 438)	**2.38**	**(2.11–2.67)**	**1.27**	**(1.10–1.46)**
**Model 2: Perinatal risk factors (*N* = 78 339, *n*_sexual offenders_ = 13 440)**						
No. of older full brothers	0.36 (s.d. = 0.67, *n* = 13 614)	0.37 (s.d. = 0.63, *n* = 68 438)	0.97	(0.95–1.00)	–	–
Low Apgar score^e^	1.3% (154/12 235)	1.2% (760/61 227)	1.02	(0.85–1.21)	–	–
Birth weight <2500 g	4.5% (621/13 773)	3.6% (2470/68 718)	**1.26**	**(1.16–1.38)**	0.97	(0.87–1.09)
SGA^f^	4.9% (660/13 453)	3.2% (2159/67 022)	**1.54**	**(1.41–1.69)**	**1.42**	**(1.29–1.57)**
Any congenital malformation	5.0% (676/13 551)	4.9% (3307/67 604)	1.01	(0.93–1.10)	–	–
Head circumference ⩽33 cm	20.5% (2822/13 773)	16.8% (11 561/68 718)	**1.29**	**(1.23–1.35)**	**1.25**	**(1.19–1.32)**

OR, Odds ratio; CI, confidence interval; aOR, adjusted odds ratio.

Mean (standard deviation, sample size) for continuous variables, % (*n*) for categorical variables. Male controls were matched 1:5 on birth year and county of birth in Sweden.

Bold values are statistically significant at *p* < 0.05. Unadjusted ORs were generated from bivariate logistic regression, not controlling for any other variables. Only statistically significant risk factors (*p* < 0.05) in bivariate analyses were entered in the final conditional model. Each aOR represents the change in the odds of committing a sexual offence for each one unit increase on the risk factor, after maintaining all other variables in the model constant.

a Sample size is reduced in the final model due to listwise deletion (i.e. participants are excluded from analysis if any single value is missing).

b Birth year was not included in analyses since it was a matching variable.

c Reference category is 25–44.9 years of age (parents aged ⩾45 were excluded from analyses).

d Highest parental education (across both biological parents): 0, primary school; 1, secondary school; 2, post-secondary qualification.

e Low Apgar: <7 at 5 min after birth.

f SGA: Small for gestational age defined as ⩾2 s.d. below the mean birth weight for gestational age. Model 1 (parental risk factors): pseudo-*R*^2^ = 0.091, log likelihood = −21 014.09, *N* = 76 636 (*n* cases = 13 168). Model 2 (perinatal risk factors): pseudo-*R*^2^ = 0.003, log likelihood = −23 549.17, *N* = 78 339 (*n* cases = 13 440).

The two final multivariable models included the 10 bivariately significant factors (Table 1; rightmost column). Adjusted odds ratios (aOR) suggested somewhat attenuated but independent, small risk effects for all seven parental factors (model 1). For the three included perinatal factors, risk effects from being small for gestational age and head circumference were both attenuated but remained independent risk factors. Small birth weight was no longer statistically significant after adjusting for the other perinatal risk factors.

### Non-sexual violent offending

Similar parental and perinatal risk factors associated with a greater likelihood of sexual offending were identified for non-sexual violent offending (Table 2). Parental factors – young parental age at offspring birth, lower parental education, any parental violent conviction, psychiatric disorder, substance use disorder, and suicide attempt – were associated with higher risk of non-sexual violent offending (small to moderate effects). Similar to sexual offending, small birth weight, being small for gestational age, and small head circumference increased non-sexual violent offending risk in bivariate analyses. Higher number of older biological brothers was also associated with non-sexual violent offending, whereas any congenital malformation appeared negatively associated.

**Table 2.** Birth year, parental, and perinatal risk factors for non-sexual violent offending in a Swedish nationwide case-control study over 37 years

Variables	% (*n*/*N*) or mean (s.d., *N*)		Unadjusted logistic regression		Adjusted logistic regression^a^	
			OR	(95% CI)	aOR	(95% CI)
	Non-sexual violent offenders	Controls				
Birth year^b^	1983 (s.d. = 6.21, *n* = 135 953)	1983 (s.d. = 6.21, *n* = 680 120)	–	–	–	–
**Model 1: Parental risk factors (*N* = 757 774, *n*_violent offenders_ = 130 887)**						
Age of mother at delivery <25 years^c^	40.9% (55 042/134 471)	27.7% (187 186/676 776)	**1.83**	**(1.81–1.86)**	**1.38**	**(1.36–1.40)**
Age of father at delivery <25 years^c^	22.4% (29 419/131 308)	13.7% (90 917/662 135)	**1.82**	**(1.80–1.85)**	**1.24**	**(1.22–1.26)**
Parental education^d^	0.89 (s.d. = 0.54, *n* = 133 951)	1.09 (s.d. = 0.56, *n* = 675 725)	**0.51**	**(0.50–0.51)**	**0.59**	**(0.58–0.60)**
Parental violent conviction	21.8% (29 335/134 366)	6.7% (45 177/676 427)	**3.94**	**(3.88–4.01)**	**3.07**	**(3.01–3.12)**
Any parental psychiatric disorder	4.3% (5846/134 557)	2.1% (14 349/677 160)	**2.12**	**(2.05–2.18)**	**1.30**	**(1.26–1.35)**
Any parental substance use disorder	3.4% (4595/134 557)	1.1% (7414/677 160)	**3.21**	**(3.09–3.33)**	**1.60**	**(1.53–1.68)**
Any parental suicide attempt	3.2% (4295/134 557)	1.3% (8672/677 160)	**2.56**	**(2.46–2.66)**	**1.50**	**(1.44–1.57)**
**Model 2: Perinatal risk factors (*N* = 738 910, *n*_violent offenders_ = 127 690)**						
No. of older biological brothers	0.37 (s.d. = 0.66, *n* = 134 557)	0.37 (s.d. = 0.63, *n* = 677 160)	**1.02**	**(1.01–1.02)**	**1.01**	**(1.005–1.02)**
Low Apgar^e^	1.2% (1406/121 478)	1.2% (7111/608 845)	0.99	(0.94–1.06)	–	–
Birth weight <2500 g	3.9% (5323/135 953)	3.6 (24 378/680 120)	**1.10**	**(1.06–1.13)**	0.98	(0.94–1.02)
SGA^f^	3.7% (4892/132 705)	3.2% (21 147/663 681)	**1.16**	**(1.13–1.20)**	**1.12**	**(1.08–1.16)**
Any congenital malformation	4.5% (5901/132 242)	4.9% (32 214/661 613)	**0.91**	**(0.88–0.94)**	**0.91**	**(0.88–0.94)**
Head circumference ⩽33 cm	18.7% (25 422/135 953)	16.8% (114 250/680 120)	**1.14**	**(1.13–1.16)**	**1.15**	**(1.13–1.17)**

OR, Odds ratio; CI, confidence interval; aOR, adjusted odds ratio.

Mean (standard deviation, sample size) for continuous variables, % (*n*) for categorical variables. Male controls were matched 1:5 on birth year and county of birth in Sweden.

Bold values are statistically significant at *p* < 0.05. Unadjusted ORs were generated from bivariate logistic regression, not controlling for any other variables. Only statistically significant risk factors (*p* < 0.05) in bivariate analyses were entered in the final conditional model. Each aOR represents the change in the odds of committing a non-sexual violent offence for each one unit increase on the risk factor, after maintaining all other variables in the model constant.

a Sample size is reduced in the final model due to listwise deletion (i.e. participants are excluded from analysis if any single value is missing).

b Birth year was not included in analyses since it was a matching variable.

c Reference category is 25–44.9 years of age (parents aged ⩾45 were excluded from analyses).

d Highest parental education (across both biological parents): 0, primary school; 1, secondary school; 2, post-secondary qualification.

e Low Apgar: <7 at 5 min after birth.

f SGA: Small for gestational age defined as ⩾2 s.d. below the mean birth weight for gestational age. Model 1 (parental risk factors): pseudo-*R*^2^ = 0.087, log likelihood = −209 506.26 *N* = 757 774 (*n* cases = 130 887). Model 2 (perinatal risk factors): pseudo-*R*^2^ = 0.001, log likelihood = −223 523.26, *N* = 738 910 (*n* cases = 127 690).

Consistent with results for sexual offending, there were independent, small to moderate risk effects on non-sexual violent offending for the seven parental variables and a strong effect of any parental violent conviction in the multivariate parental risk model (Table 2; rightmost column). Among the five perinatal risk factors (and unique to non-sexual violent offending), number of older biological brothers and any congenital malformation retained statistically significant associations. Similar to findings for sexual offenders, lower birth weight was no longer independently linked with violent offending risk and being small for gestational age exhibited a marginal association with non-sexual violent offending.

#### Sexual offender subgroups

We also examined if risk factors for sexual offending against children differed from risk factors for sexual offending against adults (Supplementary Tables S2 and S3). In the final multivariate models, parental risk factors had similar effect sizes as found for any sexual and non-sexual violent offending. Congenital malformations appeared to be a marginal risk factor for sexual offending against children, but not for sexual offending against adults. We also conducted a sensitivity analysis (Supplementary Table S4) and found that sex offenders without non-sexual violent offences had additional risk-relevant perinatal factors, similar to the sex offenders against children subanalyses. In contrast, sexual offenders with non-sexual violent offences were largely similar to the overall any sexual offence group. Parental risk factors explained more of the variance in this subgroup (pseudo-*R*^2^ = 0.15) compared to sex offenders without non-sexual violent offences (pseudo-*R*^2^ = 0.04).

#### Summary

Table 3 presents a summary of the final, adjusted models and suggested robust small to moderate independent associations with all seven parental risk factors for sexual and non-sexual violent offending alike. In contrast, the pattern of findings for perinatal risk factors was less consistent and effect sizes were smaller overall. Small for gestational age and small head circumference displayed the most robust risk effects among the six perinatal risk factors examined. Higher number of older biological brothers remained a marginal, independent risk factor only for non-sexual violence. Any congenital malformation appeared to marginally increase the risk of sexual offending against children and decrease non-sexual violent offending risk.

**Table 3.** Summary of final multivariable logistic regression models of parental and perinatal risk factors for criminal offending in a Swedish nationwide case-control study over 37 years

	Any sexual offending		Sex offenders against Children		Sex offenders against Adults		Non-sexual violent offenders	
	aOR	(95% CI)	aOR	(95% CI)	aOR	(95% CI)	aOR	(95% CI)
**Model 1: Parental risk factors**								
Age of mother at delivery <25 years^a^	**1.42**	**(1.36–1.49)** ^**a**^ ^,^ ^**b**^	**1.51**	**(1.41–1.62)** ^**a**^	**1.44**	**(1.36–1.52]** ^**a**^ ^**,**^ ^**b**^	**1.38**	**(1.36–1.40** ^**b**^
Age of father at delivery <25 years^a^	**1.24**	**(1.17–1.31)** ^**a**^	**1.25**	**(1.15–1.36)** ^**a**^	**1.25**	**(1.16–1.34)** ^**a**^	**1.24**	**(1.22–1.26)** ^**a**^
Parental education^b^	**0.56**	**(0.54–0.58)** ^**a**^ ^,^ ^**b**^	**0.52**	**(0.49–0.55)** ^**a**^	**0.55**	**(0.53–0.58)** ^**a**^ ^,^ ^**b**^	**0.59**	**(0.58–0.60)** ^**b**^
Parental violent conviction	**2.97**	**(2.80–3.14)** ^**a**^	**3.21**	**(2.96–3.48)** ^**a**^	**3.03**	**(2.82–3.25)** ^**a**^	**3.07**	**(3.01–3.12)** ^**a**^
Any parental psychiatric disorder	**1.53**	**(1.36–1.72)** ^**a**^	**1.46**	**(1.23–1.73)** ^**a**^	**1.34**	**(1.15–1.55)** ^**a**^	**1.30**	**(1.26–1.35)** ^**a**^
Any parental substance use disorder	**1.64**	**(1.42–1.91)** ^**a**^	**1.55**	**(1.26–1.91)** ^**a**^	**1.74**	**(1.45, 2.09)** ^**a**^	**1.60**	**(1.53–1.68)** ^**a**^
Any parental suicide attempt	**1.27**	**(1.10–1.46)** ^**a**^	**1.30**	**(1.06–1.58)** ^**a**^	**1.47**	**(1.22, 1.76)** ^**a**^	**1.50**	**(1.44–1.57)** ^**a**^
**Model 2: Perinatal risk factors**								
No. of older biological brothers	–	–	–	–	–	–	**1.01**	**(1.005–1.02)**
Low Apgar^c^	–	–	–	–	–	–	–	–
Birth weight <2500 g	0.97	(0.87–1.09)^a^	1.00	(0.86–1.18)^a^	0.95	(0.82–1.10)^a^	0.98	(0.94–1.02)^a^
SGA^d^	**1.42**	**(1.29–1.57)** ^**a**^	**1.51**	**(1.31–1.74)** ^**a**^	**1.24**	**(1.09–1.41)** ^**a**^ ^,^ ^**b**^	**1.12**	**(1.08–1.16)** ^**b**^
Any congenital malformation	–	–	**1.15**	**(1.02–1.30)** ^**a**^	–	–	**0.91**	**(0.88–0.94)** ^**b**^
Head circumference ⩽33 cm	**1.25**	**(1.19–1.32)** ^**a**^	**1.28**	**(1.19–1.38)** ^**a**^	**1.24**	**(1.17–1.32)** ^**a**^ ^,^ ^**b**^	**1.15**	**(1.13–1.17)** ^**b**^
Model 1 sample size (*n*_cases_/N)	13 168/76 636		5966/35 207		8220/48 247		130 887/757 774	
Model 2 sample size (*n*_cases_/N)	13 440/78 339		5893/40 460		8392/57 493		127 690/738 910	

aOR, Adjusted odds ratio; CI, confidence interval.

Male controls were matched 1:5 on birth year and county of birth in Sweden.

Bolded values are statistically significant at *p* < 0.05 in their respective final adjusted model. Non-overlapping 95% confidence intervals represents a statistically significant difference between groups at *p* < 0.01. aORs sharing the same subscript denote that the groups were not significantly different from each other on aORs (*p* > 0.01).

a Reference category is 25–44.9 years of age (parents aged ⩾ 45 years were excluded from analyses).

b Highest parental education (across both biological parents): 0, primary school; 1, secondary school; 2, post-secondary qualification.

c Low Apgar: <7 at 5 min after birth.

d SGA: Small for gestational age defined as ⩾2 s.d. below the mean birth weight for gestational age.

## Discussion

We conducted a nationwide case-control study to identify parental and perinatal risk factors associated with an increased risk of sexual offending, based on linkage of official registries with mandatory reporting, over a period of 37 years. Consistent with past research on disruptive behaviour (Harden *et al.* 2007), substance use (Shaw *et al.* 2006), juvenile delinquency (D'Onofrio *et al.* 2009), and violent offending (Coyne *et al.* 2013*a*, *b*), sons of younger mothers had a greater risk for sexual offending than sons of older mothers. Similar to violent offending (Kuja-Halkola *et al.* 2012), sons of young fathers (<25 years) and older fathers (>45 years) also posed an increased risk of sexual offending in the present study. However, due to multicollinearity, only young paternal age was included in the analyses.

Risk factors that were associated with a greater risk of sexual offending against children were generally similar to those factors identified for sexual offending against adults, with the possible exception that any congenital malformation had a small effect for sexual offending against children, but not against adults. While this specific result agrees with retrospective research suggesting that prenatal factors may be implicated in paedophilia and/or sexual offending against children (Baumbach, 2002; Cantor *et al.* 2005), two other tested perinatal factors – number of older biological brothers and low Apgar score – were not associated with sexual offending against children in bivariate analyses (Supplementary Table S3). Finally, the other perinatal risk factor (low birth weight) was bivariately significant (*p* < 0.05), but not independently linked to child sexual offending in the final multivariate model.

Identifying shared and unique parental and perinatal risk factors for sexual and non-sexual violent offending furthers our theoretical understanding and may even inform prevention efforts. Despite the generally low rates of poor offspring health (e.g. 5% of sex offenders had low birth weight), programmes that further improve perinatal care would potentially have important implications for future development of the offspring, not just in respect to sexual and violent offending, but also likely many other adverse life outcomes (Shaw *et al.* 2006; D'Onofrio *et al.* 2009). Interventions targeting these shared parental and perinatal factors could potentially reduce both non-sexual and sexual violence, in addition to other (intended) benefits. Shared risk factors (i.e. common to sexual and non-sexual violent offending) likely reflect general antisocial tendencies involving impulsivity, emotional instability, and aggressiveness (Seto, 2008). The current findings also raise the question whether parental risk factors should be included in offender risk assessment (Fazel *et al.* 2009), which would require studies showing that these risk factors improve meaningfully on existing risk assessment instruments that emphasize the offender's personal history.

Risk factors specific to sexual offending, possibly related to the development of sexual preoccupation and paraphilias, were not demonstrated in this study. Speculatively, sexual offending-specific factors might be detected solely among sex offenders who are hypersexual or paraphilic (Kjellgren *et al.* 2010; however, see also Baur *et al.* 2016). Such sexual offenders would be more likely to have different aetiologies compared to sexual offenders who are not sexually atypical but are instead similar to non-sexual violent offenders regarding their antisocial/criminal tendencies.

Of note, prior studies addressed clinical samples of paedophilic sex offenders, whereas we studied all sex offenders with child victims, not all of whom would be expected to be paedophilic (Seto, 2008). In addition, more than half of the sexual offenders had a conviction for a non-sexual violent offence. Although parental factors were largely similar for sex offenders with and without non-sexual violent offences, sex offenders without non-sexual violent offences had additional perinatal risk factors, similar to the sex offenders against children subanalyses. In contrast, sexual offenders with non-sexual violent offences were largely similar to the overall sexual offending group. Further studies specifically sampling paedophilic sex offenders without non-sexual violent offences would be informative. If relevant diagnoses are not available, offence characteristics such as having boy victims, multiple child victims, younger child victims, and unrelated child victims, can be used to select offenders who are more likely to be paedophilic (Seto & Lalumière, 2001; Seto *et al.* 2015). Large enough samples, however, may be difficult to obtain, even in nationwide register-based population-based studies (Fazel *et al.* 2012).

A well-controlled population study, also based on linked longitudinal Swedish register data, suggested that having an older brother, especially when he was close in age, was a moderate risk factor for any violent offending (Kendler *et al.* 2014). Cross-sectional studies of selected forensic samples also suggest that paedophilic offenders are more likely to have older brothers than non-paedophilic offenders (small to moderate effects; Blanchard *et al.* 2000; Côté *et al.* 2002). This contrasts with our findings that suggested that more older brothers was a (weak) risk factor for the onset of non-sexual violent offending, but not sexual offending. In fact, among sexual offenders without any non-sexual violent offences, we found that having older brothers was associated with a lower likelihood of sexual offending. It remains possible that having older brothers is risk-relevant only for paedophilia and not for sexual offending against children or sexual offending in general.

There are a number of limitations with this study. First, we examined risk factors for convictions of sexual and non-sexual violent offences, which represent a small proportion of all offences committed. It is estimated that up to 80% of all sexual offences are never reported to the police (Swedish Council on Health Technology Assessment, 2011) and many reported sexual assaults do not result in criminal charges or convictions (Swedish National Council for Crime Prevention, 2014). As such, identified risk factors may not only reflect the liability to commit an offence but also characteristics that increase the probability of being arrested and convicted as a consequence. However, although conviction data are likely to include more egregious offending (e.g. resulting in physical injury to the victim), they are also less affected by self-report biases, and allow for register linkage and international comparisons of findings.

Second, sexual offending is substantially less common than non-sexual violent offending; hence, our study's statistical power remained limited for subgroup analyses, despite using a complete national sample of sexual offenders identified over a 37-year period. The pseudo-*R*^2^ of the final models were low, though higher for parental (0.087–0.104) than birth (0.001–0.005) models, suggesting that there are additional factors not included in the current study that may explain sexual and non-sexual violent behaviours. For statistical power reasons, we decided not to conduct cousin or sibling comparisons, which were previously used to estimate if associations of risk factors and criminality are explained by shared, often unmeasured, familial factors (D'Onofrio *et al.* 2011; Forsman *et al.* 2015). This type of analysis would be important to examine whether the risk factors identified were consistent with a causal inference.

We were also limited by the parental and birth factors that were available in the dataset and had to exclude some variables due to multicollinearity (e.g. older paternal age). As such, the current study is not a complete examination of all variables that have been proposed as important in prior theoretical and empirical work. Instead, the current study is part of a continuing program of research, and the current study included a large set of factors as a first step to identify distal risk factors for sexual offending.

More nuanced analyses of identified factors would be informative to fully understand the effects (e.g. Kuja-Halkola *et al.* 2012). For example, young parental age was found to be a robust risk factor that increased the risk of both sexual and non-sexual violent offending. We also found that older paternal age was also associated with an increased risk of offspring sexual criminality. Further careful analyses are required to fully partition out parental age effects.

Finally, possible differences between offender groups defined by victim age may have been attenuated by the inclusion of sexual offenders with adolescent victims; in Sweden, children are legally defined as under the age of 15 years or, occasionally, if the adult was in a position of authority and trust (e.g. teacher), under the age of 18 years. We would expect larger differences when comparing offenders against prepubescent children with offenders against adults.

## Conclusions

We used prospectively collected, linked national register data with exposures and outcomes registered as they occurred and therefore without the recall and reporting biases of retrospective surveys of parental and other early-life factors. Analyses suggested considerable concordance in the small to moderate associations between a number of parental and perinatal risk factors and later sexual and non-sexual violent offending.

## Supplementary material

For supplementary material accompanying this paper visit http://dx.doi.org/10.1017/S003329171600249X. click here to view supplementary material
