# Supplementary material for: Parental and perinatal risk factors for sexual offending in men: a nationwide case-control study
Source: Psychol Med. 2016 Oct 13;47(2):305–15. doi: 10.1017/S003329171600249X (PMC5217130; doi:10.1017/S003329171600249X)

**SUPPLEMENTARY MATERIAL**

**Supplementary Table S1**. Correlation matrix for birth year, parental, and perinatal risk factors in a Swedish nationwide case-control study over 37 years (n=76,636).

|  | **1** | **2^a^** | **3** | **4** | **5** | **6** | **7** | **8** | **9** | **10** | **11** | **12** | **13** | **14** | **15** | **16** |
| --- | --- | --- | --- | --- | --- | --- | --- | --- | --- | --- | --- | --- | --- | --- | --- | --- |
| **1. Sexual offending** | 1.00 | - | - | - | - | - | - | - | - | - | - | - | - | - | - | - |
| **2. Nonsexual violent offending** | **.710** | 1.00 | - | - | - | - | - | - | - | - | - | - | - | - | - | - |
| **3. Birth year** | .002 | .000 | 1.00 | - | - | - | - | - | - | - | - | - | - | - | - | - |
| **4. No. of older biological brothers** | -.021 | .005 | .029 | 1.00 | - | - | - | - | - | - | - | - | - | - | - | - |
| **5. Age of mother at delivery <25 yrs^a^** | .223 | .202 | -.124 | -.357 | 1.00 | - |  | - | - | - | - | - | - | - | - | - |
| **6. Age of father at delivery <25 yrs^a^** | .195 | .187 | -.112 | -.408 | **.786** | 1.00 | - | - | - | - | - | - | - | - | - | - |
| **7. Highest parental education^b^** | -.259 | -.238 | .063 | -.027 | -.324 | -.269 | 1.00 | - | - | - | - | - | - | - | - | - |
| **8. Any parental violent conviction** | .393 | .402 | .114 | -.069 | .261 | .229 | -.264 | 1.00 | - | - | - | - | - | - | - | - |
| **9. Any parental psychiatric disorder** | .198 | .181 | .161 | -.018 | -.026 | -.018 | -.109 | .340 | 1.00 | - | - | - | - | - | - | - |
| **10. Any parental substance use disorder** | .287 | .270 | .130 | -.081 | .037 | -.040 | -.194 | .557 | .716 | 1.00 |  | - | - | - | - | - |
| **11. Any parental suicide attempt** | .201 | .217 | .203 | -.129 | .071 | .038 | -.122 | .419 | .652 | .625 | 1.00 | - | - | - | - | - |
| **12. Low Apgar score^c^** | -.002 | -.002 | -.092 | -.083 | .078 | .089 | -.068 | .035 | -.001 | .061 | .087 | 1.00 |  | - | - | - |
| **13. Birth weight <2,500 g** | .058 | .023 | .001 | -.050 | .048 | .070 | -.077 | .107 | .082 | .169 | .141 | .378 | 1.00 | - | - | - |
| **14. SGA^d^** | .107 | .037 | -.101 | -.100 | .079 | .042 | -.092 | .110 | .056 | .121 | .071 | .230 | **.770** | 1.00 |  | - |
| **15. Any congenital malformation** | .010 | -.023 | -.044 | -.021 | -.088 | .001 | .011 | -.027 | .025 | .013 | .019 | .037 | .133 | .109 | 1.00 | - |
| **16. Head circumference ≤33cm** | .077 | .041 | -.027 | -.081 | .104 | .094 | -.053 | .101 | .059 | .093 | .066 | .087 | .678 | .494 | .036 | 1.00 |

***Note.*** Tetrachoric (dichotomous variables), polychoric (non-dichotomous categorical variables, or dichotomous vs. categorical variables), and Pearson correlations (continuous variables; Kolenikov & Angeles, 2004). Correlations above ±.70 are bolded. Correlations between violent offending and variables in column 2 is based on *n*=738,910 whereas the remaining cells are based on *n*=76,636. ^a^Reference category is 25-44.9 years of age (parents aged >45 were excluded from analyses). ^b^Highest parental education (across both biological parents): 0=primary school, 1=secondary school, 2=post-secondary qualification. ^c^Low Apgar: <7 at 5 minutes after birth. ^d^SGA: Small for gestational age defined as >2 SD below the mean birth weight for gestational age.

**Supplementary Table S2.** Birth year, parental, and perinatal risk factors for *sexual offending* *against children* in a Swedish nationwide case-control study over 37 years.

| **Variables** | **Sex Offenders Against Children** | **Controls** | **Unadjusted Logistic Regression** | | **Adjusted Logistic Regression**^b^ | |
| --- | --- | --- | --- | --- | --- | --- |
|  |  |  | **OR** | **[95% CI]** | **AOR** | **[95% CI]** |
|  | **% (*n*/*N*) or *M* (*SD*, *N*)** | |  |  |  |  |
| **Birth year^a^** | 1984 (*SD*=6.43, *n*=6,263) | 1984 (*SD*=6.43, *n*=37,513) | - |  |  |  |
| **Model 1: Parental Risk Factor (*N***=**35,207, *n*_sex offenders_=5,966)** | |  |  |  |  |  |
| Age of mother at delivery <25 yrs^c^ | 43.0% (2,661/6,182) | 27.4% (8,547/31,180) | **2.02** | **[1.90, 2.13]** | **1.51** | **[1.41, 1.62]** |
| Age of father at delivery <25 yrs^c^ | 23.3% (1,391/5,981) | 13.4% (4,099/30,479) | **1.96** | **[1.83, 2.10]** | **1.25** | **[1.15, 1.36]** |
| Highest parental education^d^ | 0.85 (*SD*=0.52, *n*=6,152) | 1.06 (*SD*=0.55, *n*=37,274) | **0.49** | **[0.46, 0.52]** | **0.52** | **[0.49, 0.55]** |
| Any parental violent conviction | 23.4% (1,445/6,170) | 8.8% (3,289/37,312 | **3.18** | **[2.97, 3.41]** | **3.21** | **[2.96, 3.48]** |
| Any parental psychiatric disorder | 5.4% (334/6,184) | 2.3% (712/31,200) | **2.46** | **[2.15, 2.81]** | **1.46** | **[1.23, 1.73]** |
| Any parental substance use disorder | 3.9% (242/6,184) | 1.2% (365/31,200) | **3.43** | **[2.91, 4.06]** | **1.55** | **[1.26, 1.91]** |
| Any parental suicide attempt | 3.6% (222/6,184) | 1.4% (434/31,200) | **2.63** | **[2.23, 3.10]** | **1.30** | **[1.06, 1.58]** |
| **Model 2: Perinatal Risk Factor (*N*=40,460, *n*_sex offenders_=5,893)** | |  |  |  |  |  |
| No. of older biological brothers | 0.35 (*SD*=0.65, *n*=6,184) | 0.37 (*SD*=0.63, *n*=31,200) | 0.96 | [0.92, 1.01] | - | - |
| Low Apgar^e^ | 1.2% (66,5,633) | 1.2% (389/33,744) | 1.01 | [0.77, 1.31] | - | - |
| Birth weight < 2,500 grams | 5.0% (313/6,263) | 3.8 (1,414/37,513) | **1.34** | **[1.18, 1.52]** | 1.00 | [0.86, 1.18] |
| SGA^f^ | 5.4% (329/6,117) | 3.3% (1,198/36,570) | **1.68** | **[1.48, 1.90]** | **1.51** | **[1.31, 1.74]** |
| Any congenital malformation | 5.4% (326/6,035) | 4.7% (1,714/36,142) | **1.15** | **[1.01, 1.30]** | **1.15** | **[1.02, 1.30]** |
| Head circumference ≤ 33cm | 21.1% (1,322/6,263) | 16.7% (6,271/37,513) | **1.35** | **[1.26, 1.44]** | **1.28** | **[1.19, 1.38]** |

***Notes***. Mean (standard deviation, sample size) for continuous variables, % (*n*) for categorical variables. Male controls were matched 1:5 on birth year and county of birth in Sweden. Bolded values are statistically significant at *p*<.05. Unadjusted odds ratios (OR) were generated from bivariate logistic regression, not controlling for any other variables. Only statistically significant risk factors (*p*<.05) in bivariate analyses were entered in the final conditional model. Each AOR represents the change in the odds of committing a sexual offence for each one unit increase on the risk factor, after maintaining all other variables in the model constant. ^a^Birth year was not included in analyses since it was a matching variable. ^b^Sample size is reduced in the final model due to listwise deletion (i.e., participants are excluded from analysis if any single value is missing). ^c^Reference category is 25-44.9 years of age (parents aged >45 were excluded from analyses). ^d^Highest parental education (across both biological parents): 0=primary school, 1=secondary school, 2=post-secondary qualification. ^e^Low Apgar: <7 at 5 minutes after birth. ^f^SGA: Small for gestational age defined as >2 SD below the mean birth weight for gestational age. Model 1 (parental risk factors): pseudo *R^2^* =.104, Log likelihood=-9,767.24, *N*=35,207 (*n* cases=5,966). Model 2 (perinatal risk factors): pseudo *R^2^* =.005, Log likelihood=-11,633.80, *N*=40,460 (*n* cases=5,893).

**Supplementary Table S3.** Birth year, parental, and perinatal risk factors for *sexual offending against an adult* (*n*=8,220) in a Swedish nationwide case-control study over 37 years.

| **Variables** | **Sex Offenders Against Adults** | **Controls** | **Unadjusted Logistic Regression** | | **Adjusted Logistic Regression**^b^ | |
| --- | --- | --- | --- | --- | --- | --- |
|  |  |  | **OR** | **[95% CI]** | **AOR** | **[95% CI]** |
|  | **% (*n*/*N*) or *M* (*SD*, *N*)** | |  |  |  |  |
| **Birth year^a^** | 1982 (*SD*=5.77, *n*=8,584) | 1982 (*SD*=5.77, *n*=51,417) | - |  |  | - |
| **Model 1: Parental Risk Factors (*N*=48,247, *n*_sex offenders_=8,220)** | |  |  |  |  |  |
| Age of mother at delivery <25 yrs^c^ | 42.6% (3,617/8,480) | 28.2% (12,039/42,719) | **1.91** | **[1.82, 2.00]** | **1.44** | **[1.36, 1.52]** |
| Age of father at delivery <25 yrs^c^ | 23.2% (1,916/8,250) | 13.9% (5,835/41,854) | **1.85** | **[1.78, 2.00]** | **1.25** | **[1.16, 1.34]** |
| Parental education^d^ | 0.86 (*SD*=0.54, *n*=8,440) | 1.06 (*SD*=0.56, *n*=51,052) | **0.51** | **[0.49, 0.54]** | **0.55** | **[0.53, 0.58]** |
| Any parental violent conviction | 22.3% (1,887/8,472) | 8.4% (4,295/51,120) | **3.13** | **[2.94, 3.32]** | **3.03** | **[2.82, 3.25]** |
| Any parental psychiatric disorder | 4.4% (376/8,485) | 2.1% (881/42,745) | **2.21** | **[1.96, 2.50]** | **1.34** | **[1.15, 1.55]** |
| Any parental substance use disorder | 3.6% (306/8,485) | 1.1% (453/42,745) | **3.52** | **[3.03, 4.08]** | **1.74** | **[1.45, 2.09]** |
| Any parental suicide attempt | 3.0% (250/8,485) | 1.2% (505/42,745) | **2.55** | **[2.18, 2.97]** | **1.47** | **[1.22, 1.76]** |
| **Model 2: Perinatal Risk Factors (*N*=57,493, *n*_sex offenders_=8,392)** | |  |  |  |  |  |
| No. of older biological brothers | 0.37 (*SD*=0.67, *n*=8,485) | 0.36 (*SD*=0.62, *n*=42,745) | 1.01 | [0.97, 1.04] | - | - |
| Low Apgar^e^ | 1.3% (97/7,630) | 1.3% (576/45,532) | 0.99 | [0.79, 1.23] | - | - |
| Birth weight <2,500 grams | 4.2% (365/8,584) | 3.7% (1,905/51,417) | **1.15** | **[1.03, 1.29]** | 0.95 | [0.82, 1.10] |
| SGA^f^ | 4.4% (370/8,392) | 3.3% (1,672/50,220) | **1.33** | **[1.18, 1.49]** | **1.24** | **[1.09, 1.41]** |
| Any congenital malformation | 4.8% (409/8,493) | 4.8% (2,444/50,855) | 0.99 | [0.89, 1.11] | - | - |
| Head circumference ≤33cm | 20.7% (1,775/8,584) | 17.4% (8,933/51,417) | **1.25** | **[1.18, 1.33]** | **1.24** | **[1.17, 1.32]** |

***Notes***. Mean (standard deviation, sample size) for continuous variables, % (*n*) for categorical variables. Male controls were matched 1:5 on birth year and county of birth in Sweden. Bolded values are statistically significant at *p*<.05. Unadjusted odds ratios (OR) were generated from bivariate logistic regression, not controlling for any other variables. Only statistically significant risk factors (*p*<.05) in bivariate analyses were entered in the final conditional model. Each AOR represents the change in the odds of committing a sexual offence for each one unit increase on the risk factor, after maintaining all other variables in the model constant. ^a^Birth year was not included in analyses since it was a matching variable. ^b^Sample size is reduced in the final model due to listwise deletion (i.e., participants are excluded from analysis if any single value is missing). ^c^Reference category is 25-44.9 years of age (parents aged >45 were excluded from analyses). ^d^Highest parental education (across both biological parents): 0=primary school, 1=secondary school, 2=post-secondary qualification. ^e^Low Apgar: <7 at 5 minutes after birth. ^f^SGA: Small for gestational age defined as >2 SD below the mean birth weight for gestational age. Model 1 (parental risk factors): pseudo *R^2^* =.096, Log likelihood=-13,139.34, *N*=48,247 (*n* cases=8,220). Model 2 (perinatal risk factors): pseudo *R^2^* =.002, Log likelihood=-16,100.44, *N*=57,493 (*n* cases=8,392).

**Supplementary Table S4.** Parental and perinatal risk factors for *any sexual offending* stratified by history of nonsexual violent offences.

|  | **Sex Offenders** | | | | | | **Complete Nonsexual Violent Sample** | |
| --- | --- | --- | --- | --- | --- | --- | --- | --- |
|  | **Complete Sex Offender Sample** | | **Sex Offenders without Nonsexual Violent Offending** | | **Sex Offender with Nonsexual Violent Offending** | |  |  |
|  | **AOR** | **[95% CI]** | **AOR** | **[95% CI]** | **AOR** | **[95% CI]** | **AOR** | **[95% CI]** |
| **Model 1: Parental Risk Factors** |  |  |  |  |  |  |  |  |
| Age of mother at delivery <25 yrs^1^ | **1.42** | **[1.36, 1.49]** | **1.37** | **[1.28, 1.46]** | **1.48** | **[1.38, 1.58]** | **1.38** | **[1.36, 1.40]** |
| Age of father at delivery <25 yrs^1^ | **1.24** | **[1.17, 1.31]** | **1.09** | **[1.005, 1.19]** | **1.36** | **[1.26, 1.47]** | **1.24** | **[1.22, 1.26]** |
| Parental education^2^ | **0.56** | **[0.54, 0.58]** | **0.66** | **[0.62, .70]** | **0.49** | **[0.46, 0.51]** | **0.59** | **[0.58, 0.60]** |
| Parental violent conviction | **2.97** | **[2.80, 3.14]** | **1.84** | **[1.68, 2.02]** | **4.06** | **[3.77, 4.37]** | **3.07** | **[3.01, 3.12]** |
| Any parental psychiatric disorder | **1.53** | **[1.36, 1.72]** | **1.34** | **[1.11, 1.61]** | **1.70** | **[1.45, 1.98]** | **1.30** | **[1.26, 1.35]** |
| Any parental substance use disorder | **1.64** | **[1.42, 1.91]** | **1.29** | **[1.01, 1.64]** | **1.90** | **[1.57, 2.30]** | **1.60** | **[1.53, 1.68]** |
| Any parental suicide attempt | **1.27** | **[1.10, 1.46]** | 1.20 | [0.96, 1.50] | **1.30** | **[1.08, 1.57]** | **1.50** | **[1.44, 1.57]** |
| **Model 2: Perinatal Risk Factors** |  |  |  |  |  |  |  |  |
| No. of older biological brothers | **-** | - | **0.93** | **[0.89, 0.97]** | - | - | **1.01** | **[1.005, 1.02]** |
| Low Apgar^3^ | - | - | - | - | - | - | - | - |
| Birth weight <2,500 g | 0.97 | [0.87, 1.09] | 0.89 | [0.75, 1.06] | 1.04 | [0.89, 1.21] | 0.98 | [0.94, 1.02] |
| SGA^4^ | **1.42** | **[1.29, 1.57]** | **1.46** | **[1.25, 1.70]** | **1.40** | **[1.22, 1.60]** | **1.12** | **[1.08, 1.16]** |
| Any congenital malformation | - | - | **1.14** | **[1.01, 1.30]** | - | - | **0.91** | **[0.88, 0.94]** |
| Head circumference ≤33cm | **1.25** | **[1.19, 1.32]** | **1.20** | **[1.11, 1.29]** | **1.29** | **[1.20, 1.38]** | **1.15** | **[1.13, 1.17]** |
| **Model 1 Sample size (*n*_cases_/N)** | 13,168/76,636 | | 5,924/34,478 | | 7,244/42,158 | | 130,887/757,774 | |
| **Model 2 Sample size (*n*_cases_/N)** | 13,440/78,339 | | 5,832/33,852 | | 7,434/35,959 | | 127,690/738,910 | |

***Notes.*** Complete sample of sex offenders: Model 1 (parental risk factors): pseudo *R^2^* =.091, Log likelihood=-21,014.09, *N*=76,636 (*n* cases=13,168). Model 2 (perinatal risk factors): pseudo *R^2^* =.003, Log likelihood=-23,549.17, *N*=78,339 (*n* cases=13,440). Sex offenders without nonsexual violence: Model 1 (parental risk factors): pseudo *R^2^* =.036, Log likelihood=-10,031.66, *N*=34,478 (*n* cases=5,924). Model 2 (perinatal risk factors): pseudo *R^2^* =.003, Log likelihood=-10,191.92, *N*=33,852 (*n* cases=5,832). Sex offenders with nonsexual violence: Model 1 (parental risk factors): pseudo *R^2^* =.151, Log likelihood=-10,800.76, *N*=42,158 (*n* cases=7,244). Model 2 (perinatal risk factors): pseudo *R^2^* =.004, Log likelihood=-13,028.34, *N*=35,959 (*n* cases=7,434). Nonsexual violent offenders: Model 1 (parental risk factors): pseudo *R^2^* =.087, Log likelihood=-209,506.26 *N*=757,774 (*n* cases=130,887). Model 2 (perinatal risk factors): pseudo *R^2^* =.001, Log likelihood=-223,523.26, *N*=738,910 (*n* cases=127,690).

**Supplementary Figure S1**. Parental age as a risk factor for the onset of sexual offending in sons. There were curvilinear relationships between both paternal and maternal age and offspring sexual offending. There are no cases in the ≥45 years maternal age bin because of small sample size.


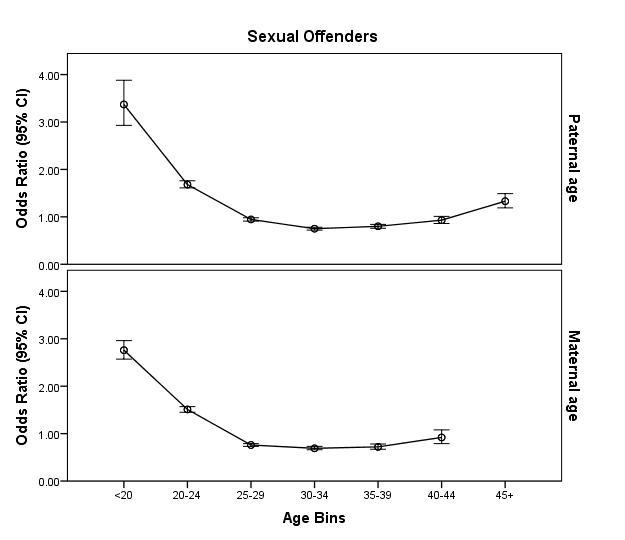


**Supplementary Figure S2**. Parental age as a risk factor for the onset of nonsexual violent offending in sons. There were curvilinear relationships between both paternal and maternal age and offspring violent offending. There are no cases in the ≥45 years maternal age bin.


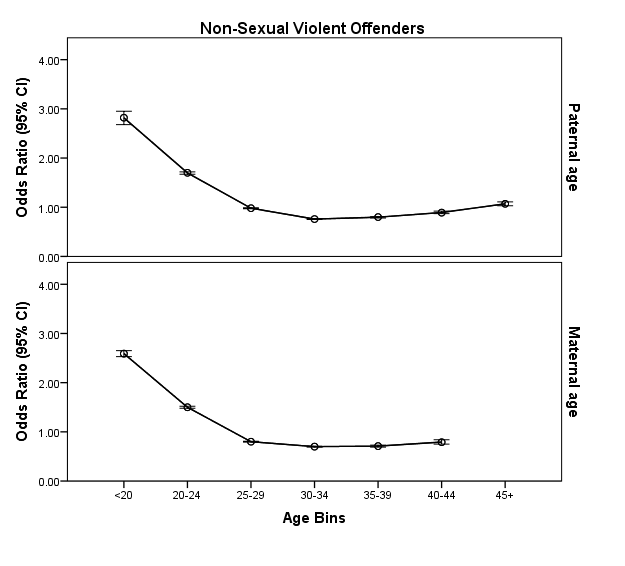

Supplement: Supplementary file 1 [file S003329171600249Xsup001.docx]
